# Variations in Author Gender in Obstetrics Disease Prevalence Literature: A Systematic Review

**DOI:** 10.3390/ijerph20010727

**Published:** 2022-12-30

**Authors:** María Rosario Román Gálvez, Blanca Riquelme-Gallego, María del Carmen Segovia-García, Daniel Gavilán-Cabello, Khalid Saeed Khan, Aurora Bueno-Cavanillas

**Affiliations:** 1 Departamento de Enfermería, Facultad de Ciencias de la Salud, Universidad de Granada, 18071 Granada, Spain; 2 Unidad Asistencial Churriana de la Vega, Servicio Andaluz de Salud, Churriana de la Vega, 18194 Granada, Spain; 3 Instituto de Investigación Biosanitaria de Granada ibs, 18014 Granada, Spain; 4 Departamento de Medicina Preventiva y Salud Pública, Facultad de Medicina, Universidad de Granada, 18071 Granada, Spain; 5 Departamento de Estadística e Investigación Operativa, Universidad de Granada, 18071 Granada, Spain; 6 Consortium for Biomedical Research in Epidemiology and Public Health (CIBERESP), 28029 Madrid, Spain

**Keywords:** gender gap, intimate partner violence, research, authorship, leadership, publications

## Abstract

This systematic review aims to evaluate gender differences in authorship of prevalence literature concerning intimate partner violence (IPV) during pregnancy and gestational diabetes mellitus (GDM). GDM studies were matched for publication year and study country as a gender-neutral obstetric disease with similar morbidity to IPV. Relevant studies were captured without language restrictions via online searches of PubMed, Scopus and Web of Science from database inception to January 2022. Proportion of female authors and gender of the first and corresponding author were outcome measures. Multivariable regression models were built to examine if female authors featured more or less often in IPV during pregnancy and GDM literature adjusting by the influence of type of study, country’s human development index (HDI), year of publication and journal’s impact factor. 137 IPV-GDM studies pairs were included. Female authors in IPV studies were slightly lower than in GDM [59.7%, 95% CI 54.7–64.7, vs. 54.9%, 95% CI 50.7–59.1, *p* = 0.204]. Studies published in high-income countries were more likely to be signed by a woman as first and corresponding author (Odds Ratio 2.22, 95% CI 1.20; 4.11, *p* = 0.011 and Odds Ratio 2.24, CI 1.22; 4.10, *p* = 0.009 respectively) and proportion of women as corresponding authors decreased as the journal impact factor increased (β = 0.62, 95% CI 0.37, 1.05, *p* = 0.075). There is a gender gap in the field of prevalence research in IPV during pregnancy with variations according to the level of development. International programs aimed at eradicating these inequalities are needed.

## 1. Introduction

Women represent 70% of the global health workforce, yet today there is a gender inequity in the academic health sector [1], however, less than two-fifths of senior academics (professors, deans, presidents and senior university leaders) and less than one-third of research authors are women in the academic health sector, so, as in other academic fields, women tend to be under-represented [2]. Women are under-represented in research careers, as they are in many other sectors. Only 30% of corresponding authors are women, suggesting that female researchers may have less opportunity to both enter and advance in their fields [1]. Gender gap in research has been previously demonstrated in fields of oncology [3,4], cardiology [5,6], hepatology [7], intensive care [8], nursing journals [9] and epidemiology [10] among others. However, it is unknown whether these inequities also exist in obstetrics, particularly in an area such as intimate partner violence (IPV) research.

The extent to which gender disparity in authorship specifically affects research in gender violence is important because IPV is a condition that is predominantly perpetrated by men against women. This background may affect the level of interest men and women researchers show in this subject. For comparative analysis, a study would need to deploy a gender-neutral disease that affects the same life period with a similar morbidity level as a control condition. The level of morbidity due to IPV during pregnancy is similar to that of gestational diabetes mellitus (GDM), and the latter meets the gender-neutrality criterion in that it is not a condition where gender has an influence. Both IPV during pregnancy and GDM are problems typically without obvious symptomatology and with an adverse impact on pregnancy outcome [11,12]. Antenatal care deploys screening to detect both conditions. With the above background, we hypothesized that there is a gender gap in the authorship of worldwide prevalence studies of IPV during pregnancy and GDM [13,14]. We aimed to compare the gender of authorship of published prevalence literature on IPV during pregnancy with that of GDM prevalence studies using multivariable models accounting for the impact of country development status and the journal impact factor.

## 2. Materials and Methods

This systematic review was carried out following prospective registration (ID: blinded).

### 2.1. Literature Search and Selection of IPV during Pregnancy and GDM Prevalence Studies

Studies concerning IPV during pregnancy were selected from a previous review reporting the worldwide prevalence of IPV in pregnant women (n = 155) [15]. The GDM prevalence literature was searched to create a group of studies matched against the IPV during pregnancy prevalence studies. We conducted searches in PubMed, WOS and Scopus databases using the following search string combining MeSH and free text terms: ((“Gestational Diabetes” AND (“Pregnancy” OR “Pregnant Women” OR “Prenatal Care”)) AND “Prevalence”). Two reviewers (RMRG and BRG) independently selected citations and studies that met the following criteria: cross-sectional or cohort study design and that presented prevalence rates of GDM. No language or geographical restrictions were applied.

### 2.2. Matching of IPV during Pregnancy and GDM Studies

Worldwide prevalence of GDM were selected and matched with IPV during pregnancy prevalence studies (1:1) by: 1—year of publication (±5 years) and, 2—study country. When no GDM studies from the same country were identified because there were none or they were already used, they were matched with a similar study country’s HDI, seeking the smallest difference between both of them. When several countries with similar HDI were found, the geographically closest country was selected as a control. When there were several studies meeting these criteria, we chose the one published in the closest Impact Factor Journal.

### 2.3. Authorship Gender Identification

Authorship gender was identified by examining profiles on social media networks, as has been previously reported [5,6,7,8,9]. Prioritizing platforms most related to research, we collected information from sites in a hierarchical fashion starting with ResearchGate and Google Scholar, then moving to university directories, and finally to Facebook. In the remaining authors, Genderize [16], a tool to infer the gender from first names, was used. The corresponding authors were contacted by e-mail to find out the gender of the authors as a last resort.

### 2.4. Statistical Analysis

The statistical analysis compared the characteristics between the IPV and the GDM groups. All the tests in this analysis were carried out with a significance level of 0.05. The comparison was conducted by means of the Chi-square test in most of the cases, as we were dealing with categorical variables, the sole exception was the variable proportion of women, that is a numerical variable. In the latter case, given the non-normality of the data collected for this variable, the groups have been compared by means of the Wilcoxon non-parametric test. The normality of the data has been tested using the Lilliefors test. Additionally, we have considered paired samples, as each IPV study has been paired with a GMD study based on the described characteristics. Afterwards, we present univariate and multivariate regression analyses, where the main interest was predicting the gender of the author given the rest of the variables in the study, i.e., Type of study (IPV/GDM), Human Development Index, Journal Impact Factor and Year. In particular, we performed regression analyses considering each of the gender variables as a dependent variable (i.e., Proportion of female authors, First author female, Last author female and Corresponding author female). For the proportion of female authors, univariate and multivariate linear regression analyses were carried out, and for the rest of the variables, logistic regression analyses were performed. For the linear regression analyses we test the assumptions on the residuals and the non-collinearity of the variables, all the assumptions are met. However, the adjusted R-squared is very poor for all the models, univariate or multivariate, around 0.05. For the logistic regression analyses, we perform the Hosmer and Lemeshow test for the goodness of fit, and the Omnibus test to check if any of the proposed models outperformed the baseline model, that is, the one that does not contain any explanatory variables. Finally, we apply Multiple Correspondence Analysis (MCA). MCA is an extension of correspondence analysis (CA), and it is equivalent to principal component analysis (PCA), but with categorical variables instead of quantitative variables. MCA is a technique to analyse the dependence relationships between more than two categorical variables by representing these categories in a Euclidean space of low dimensions (2 or 3 dimensions, normally). When we represent these categories in a 2-dimensional space, we can interpret their relationships, e.g., two categories that are close two each other in the graphical representation are presented in the same individuals. A category or a group of categories close to the origin mean that the appear together in a bigger proportion than other categories on the collected data. Two softwares were utilised to analyse the data: IBM SPSS Statistics 28.0.1.0 and R 4.1.2.

## 3. Results

**Figure 1:**
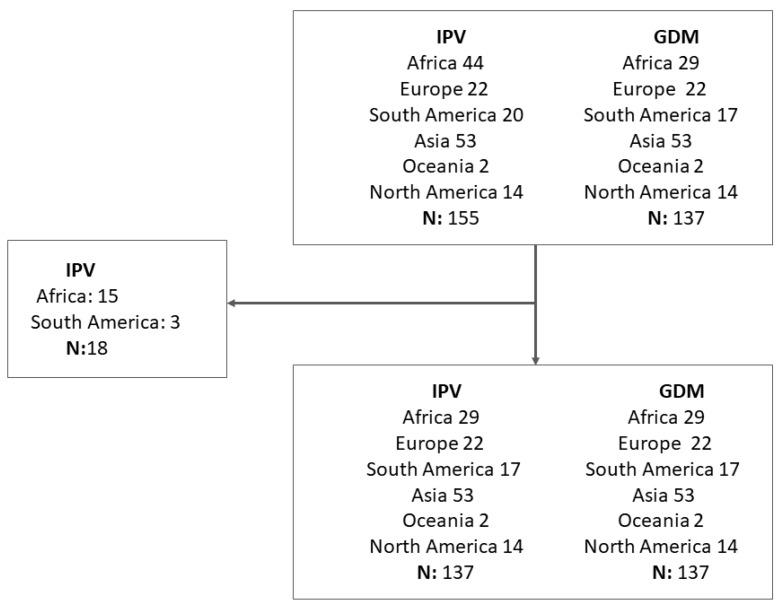
Flowchart of the prevalence studies of intimate partner violence (IPV) during pregnancy and gestational diabetes mellitus (GMD) included in the author gender study.

There were a total of 155 IPV during pregnancy studies in the published review (15). There were 137 IPV-GDM study pairs included in the final quantitative analysis (Figure 1). We did not find a suitable pair among GDM prevalence studies for 15 and 3 IPV studies coming from the African and South American regions respectively.

Table 1 shows the main characteristics of the GDM and IPV during pregnancy prevalence studies included, as well as the authorship gender. The proportion of women authors in the IPV during pregnancy prevalence studies was slightly higher than in the GDM prevalence studies, but not significant. However, it is noted that the proportion of women in a relevant authorship position is similar in both type of studies. Studies published after 2015 were slightly higher in the GDM group, although with no significant differences. Regarding the impact factor, IPV during pregnancy studies are indexed in journals with a significantly lower score than GDM prevalence studies. In addition, many of the studies of IPV prevalence during pregnancy are not indexed. Regarding HDI, no differences were observed between both types of study.

Table 2 shows the influence of the characteristics of the prevalence studies (type of study, HDI, impact factor and year of publication) on the total proportion of women in the authorship. It is observed that the higher HDI of the country where the studies are conducted, the higher the prevalence of women in the authorship (β = 0.14, 95% Confidence Interval (CI): 0.05; 0.22, *p* = 0.001). Regarding the year of publication, there is a lower proportion of women in the authorship of IPV during pregnancy and GDM prevalence studies published after 2015 (β = −0.08, CI −0.07, 0.07). However, this association was not significant when adjusted for the type of study, HDI and the impact factor.

Table 3 shows the influence of the prevalence studies’s characteristics (type of study, HDI, impact factor and year of publication) on the proportion of women as the first author in the prevalence studies analysed. As observed, studies published in a high-income country were 2.22 times more likely to be signed as a fist author by a woman than in low-income countries (OR = 2.22, CI 1.20; 4.11, *p* = 0.011), after adjusting by the influence of the other studies characteristics.

Table 4 shows the association between the proportion of studies in which the last author was a woman in IPV during pregnancy and GDM prevalence studies and the prevalence studies’s characteristics previously described. The variables that remained in the model after adjusting for the confounding variables were the type of study and the HDI, however both were non-significantly associated with the response variable.

When the proportion of women corresponding author in IPV during pregnancy and GDM prevalence studies was analysed, it was observed that variables that remained in the adjusted model were again type of study, the HDI and the impact factor of the journal. However, HDI was the most significantly associated, since it is 2.24 more likely to find a corresponding female author in those prevalence studies published in high HDI countries than in low HDI countries. Regarding the impact factor, we observed that the number of women corresponding authors decreases as the impact factor of the journals increases, although it was not significant (OR = 0.62, CI 0.37, 1.05, *p* = 0.075) (Table 5).

**Figure 2:**
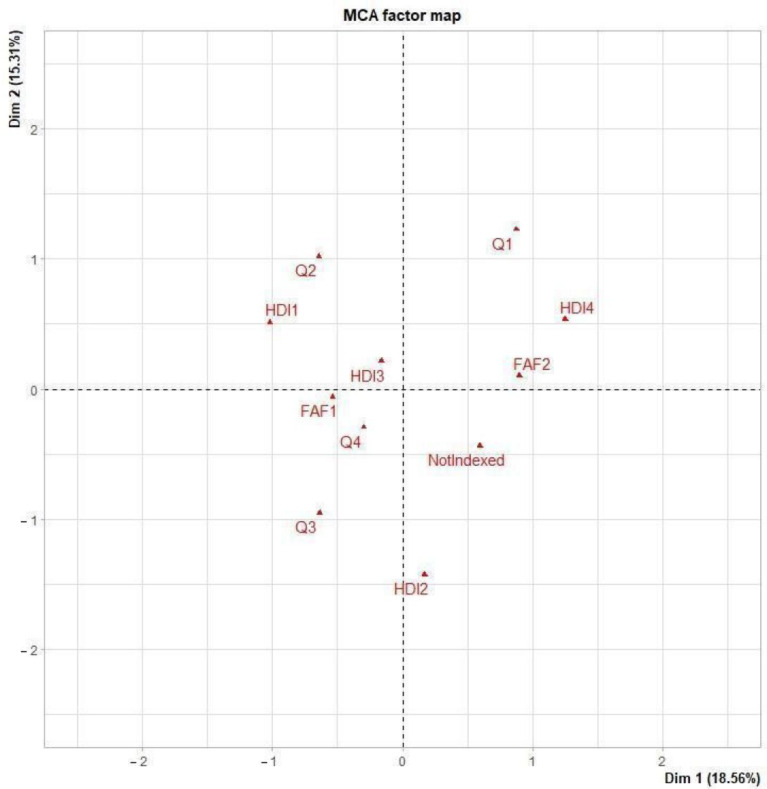
Multiple correspondence analysis for Intimate Partner Violence during pregnancy prevalence studies. The categories represented on the figures are the following: For the variable FAF: FAF1 represents that the first author is female, FAF2 represents a first author male; for the variable HDI: HDI1 is a very high HDI, HDI2 is a high HDI, HDI3 is medium HDI and HDI4 is a low HID; for the variable JIF: Q1 is a first quartile journal, Q2 is a second quartile journal, Q3 is a third quartile journal, Q4 is a fourth quartile journal, Not indexed is a not indexed journal.

**Figure 3:**
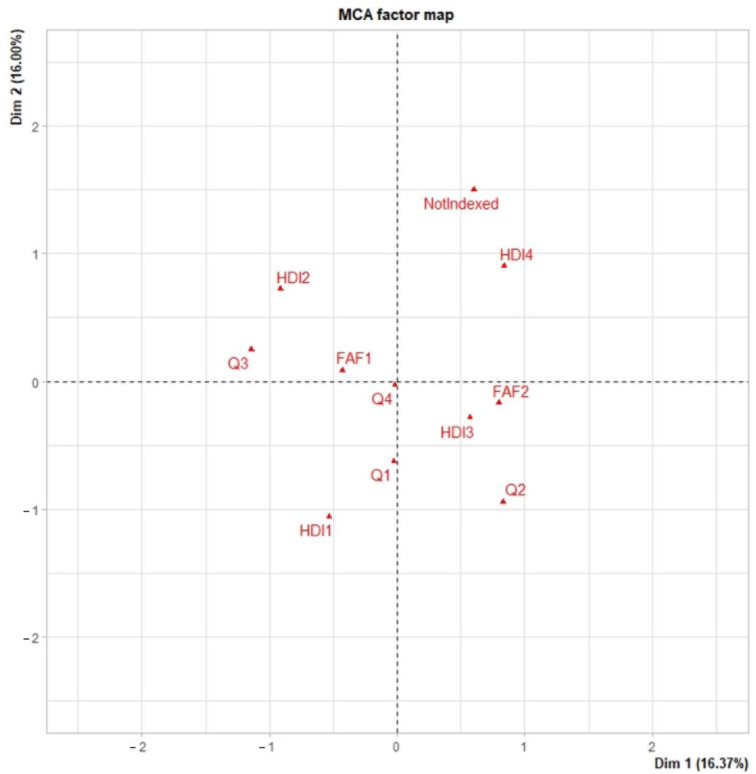
Multiple correspondence analysis for Gestational Diabetes Mellitus prevalence studies. The categories represented on the figures are the following: For the variable FAF: FAF1 represents that the first author is female, FAF2 represents a first author male; for the variable HDI: HDI1 is a very high HDI, HDI2 is a high HDI, HDI3 is medium HDI and HDI4 is a low HID; for the variable JIF: Q1 is a first quartile journal, Q2 is a second quartile journal, Q3 is a third quartile journal, Q4 is a fourth quartile journal, Not indexed is a not indexed journal.

The multiple correspondence analysis (MCA) results are presented for prevalence IPV during pregnancy studies in Figure 2 and for GDM prevalence studies in Figure 3. Both figures show the correlation between the variables categories: First Author Female (FAF), HDI and the JIF giving the type of scientific article. In Figure 2, we observe the categories that are closer to the origin, (0,0) given that those are the ones that appear in the dataset more frequently. In this graph, HDI3, FAF1 and Q4 are quite close to the origin, and that means that for an IPV article the most common situation is to have a first author female, publishing in low impact journals and a from medium HDI country. This can be confirmed by looking at the bottom half of the graph, where we can see that first author female appears on the same side of the graph to publications in journals on the third or fourth quartile, or even not indexed, that is, a first author female for IPV articles is more frequent in low impact journals. On the top half of the graph, we can see that the first male author is close to a HDI low, meaning that in these countries there is a bigger proportion of first authors that are men. Additionally, a first author male is closer to higher impact factor journals, first quartile journals are relatively correlated with having a first author male. For this type of study, it seems that publications in countries with high HDI tend to occur in second quartile journals.

In Figure 3, are shown the categories that appear more frequently together are FAF1 y Q4, again a first author female publishing in a low impact journal. We can observe as well that this type of article in low HDI countries (HDI4) tend to appear in not indexed journals, these categories appear on the right side of the graph, as well as FAF2 (first author male), therefore, the first author of GDM articles in these countries, as well as in medium HDI countries (HDI3) is more frequently a man. On the left side of the graph, we can see that a first author female for GDM articles occur more frequently in HDI1 and HD2 countries (very high and high HDI countries). Category HDI1 appears close to Q1, this means that is more frequent to have this type of articles in very high HDI countries published in high impact journals (Q1).

## 4. Discussion

Our study observed a slightly higher proportion of female authors in IPV prevalence studies during pregnancy than in GDM prevalence studies. HDI was strongly associated with the higher prevalence of total female authors and first or corresponding female authors. Prevalence studies published in a high-income country were twice as more likely to be signed by a woman as first author than studies carried out in low-income countries. Same tendency was found when gender corresponding author is analysed, since it was twice as more likely to find a woman corresponding author in high HDI countries compared to low HDI countries. This review has strengths and some limitations. Although the procedure of searching for the authors’ gender does not require extensive computer skills, it should be noted that it is difficult to find out the author’s full names. In these situations, it was considered to search in the universities’s directories and to try to find publications where their names appeared in a complete form. Research-related platforms were also used, such as ResearchGate and Google Scholar, since this strategy made it possible to find people sharing names with the same gender. The Genderize tool, considered to be one of the most accurate tools [10], was also very useful for the gender search. Nonetheless some authors remained unclassified. In addition, important variables such as the authors’s age were not studied, what has been proven as a key factor in authorship gender [17]. Nonetheless, to our knowledge, this is the first study to analyse the gender of authorship in IPV research during pregnancy.

The Lancet journal reported that in 2018, just one-third of scientific authorship was female [1]. Moreover, Cash-Gibson et al. reported that less than 6% of countries represented in the Web of Science database were close to achieving gender parity in terms of published articles [18]. If we analyse this gender gap by fields, the percentage of women was particularly low in oncology (31.7%) [3], cardiovascular research (24%) [5]; or transplantation (30.4%) [19]. Nursing research is the only field where the proportion of female researchers exceed that of male researchers [20]. Our initial hypothesis does not hold true, but some findings can be noted. Even when there is a higher proportion of females in the authorship of IPV during pregnancy comparing with another important health topic during pregnancy as GDM, there was no differences among relevant authorship. It could be because women researchers are less likely to be attributed a leader authorship than men [21]. Other studies have also agreed with our results, in which the higher proportion of women as first author is observed in studies conducted in high HDI countries [17,18,19,20,21,22,23], and there was a decreasing number of females as corresponding authors as the impact factor of journals increases [24]. We expected to find a higher percentage of women in the authorship of studies on IPV during pregnancy, since women’s health is a particular target of discrimination and its academic characteristics for social science related aspects might thus be different [25]. However, this could not be confirmed in the sample of studies selected for this review. Future research in this field should go further by collecting data on the age of authorship, as women’s research careers become more complicated compared to their peers as they get older. Nevertheless, the most significant issue is to identify effective interventions to correct, or at less compensate, this gender gap.

## 5. Conclusions

There is a gender gap in the field of prevalence research in IPV during pregnancy and GDM. This gap is particularly noticeable in senior positions, higher in less developed countries, and closely related to the impact level of the journal. This points out the need for international programmes aimed at eradicating these inequalities, taking into account the peculiarities of each region and its level of development.

## Figures and Tables

**Table 1:** Characteristics of intimate partner violence (IPV) during pregnancy and gestational diabetes mellitus (GDM) prevalence studies included in the author gender study.

	Type of Study		*p*-Value
	IPV (Total = 137)	GDM (Total = 137)	
Author gender			
Proportion of female authors, % (95% Confidence Interval)	59.7% (54.7–64.7)	54.9% (50.7–59.1)	0.204 (**)
First author female	85 (61.6%)	87(64.0%)	0.684
Last author female	73 (52.5%)	78 (57.4%)	0.420
Corresponding author female	79 (57.2%)	81 (60.0%)	0.644
Study characteristics			
Year			
<2015	71 (51.4%)	57 (41.0%)	
≥2015	67 (48.6%)	82 (59.0%)	0.081
Journal Impact Factor (quartiles)			
Not indexed	50 (35.7%)	27 (19.3%)	(*)
Q1	14 (10.0%)	37 (26.4%)	(*)
Q2	26 (18.5%)	23 (16.4%)	0.002
Q3	25 (17.9%)	27 (19.3%)	
Q4	25 (17.9%)	26 (18.6%)	
Human Development Index (HDI)			0.962
HDI1 (very high)	58 (41.4%)	62 (44.3%)	
HDI2 (high)	35 (25.0%)	32 (23.0%)	
HDI3 (medium)	25 (17.9%)	25 (17.9%)	
HDI4 (low)	22 (15.7%)	21 (15.0%)	

Data presented as n (%) unless otherwise stated; (*) There are significant differences between the proportions of these groups. (**) Non-parametric test due to no normality of the data.

**Table 2:** Multivariate regression estimates the proportion of female author gender among prevalence studies of intimate partner violence (IPV) during pregnancy and prevalence of gestational diabetes mellitus (GDM) included.

	Univariate Analysis			Multivariate Analysis		
	Parameter Estimation	95% Confidence Interval	*p*-Value	Parameter Estimation	95% Confidence Interval	*p*-Value
Dependent variable: Prop. of female author (Linear regression)						
Type of study (IPV/GDM)	−0.02	−0.07; 0.06	0.785	−0.01	−0.07; 0.06	0.869
Human Development Index (Low/high) (*)	0.22	0.07; 0.23	<0.001	0.14	0.05; 0.22	0.001
Journal impact factor (Low/high) (**)	0.01	−0.07; 0.07	0.931	0.01	−0.06; 0.08	0.792
Year (<2015/≥2015)	−0.08	−0.15; −0.02	0.015	−0.06	−0.13; 0.01	0.070

(*) Low HDI versus the rest. (**) Q3, Q4 and not indexed versus Q1 and Q2.

**Table 3:** Multivariate regression estimates of first author gender among prevalence studies of intimate partner violence (IPV) during pregnancy and prevalence of gestational diabetes mellitus (GDM) included.

	Univariate Analysis			Multivariate Analysis		
	Odds Ratio	95% Confidence Interval	*p*-Value	Adjusted Odds Ratio	95% Confidence Interval	*p*-Value
Dependent variable: First author female (Logistic regression Reference group: male)						
Type of study (Reference group: IPV)	1.12	0.67; 1.85	0.672	1.17	0.69; 1.97	0.561
Human Development Index (Reference group: Low HDI) (*)	2.30	1.26; 4.19	0.007	2.22	1.20; 4.11	0.011
Journal impact factor (Reference group: Low JIF) (**)	0.76	0.45; 1.27	0.295	0.74	0.43; 1.27	0.272
Year (Reference group: <2015)	0.69	0.42; 1.15	0.158	0.80	0.47; 1.37	0.415

(*) Low HDI versus the rest. (**) Q3, Q4 and not indexed versus Q1 and Q2.

**Table 4:** Multivariate regression estimates of last author gender among prevalence studies of intimate partner violence (IPV) during pregnancy and prevalence of gestational diabetes mellitus (GDM) included.

	Univariate Analysis			Multivariate Analysis		
	Odds Ratio	95% Confidence Interval	*p*-Value	Adjusted Odds Ratio	95% Confidence Interval	*p*-Value
Dependent variable: Last author female (Logistic regression Reference group: male)						
Type of study (Reference group: IPV)	1.37	0.84; 2.23	0.208	1.37	0.84; 2.23	0.214
Human Development Index (Reference group: Low HDI) (*)	1.50	0.82; 2.71	0.181	1.50	0.82; 2.71	0.186
Journal impact factor (Reference group: Low JIF) (**)	1.08	0.65; 1.79	0.769	-	-	-
Year (Reference group: <2015)	0.83	0.51; 1.36	0.456	-	-	-

(*) Low HDI versus the rest. (**) Q3, Q4 and not indexed versus Q1 and Q2.

**Table 5:** Multivariate regression estimates of corresponding author gender among prevalence studies of intimate partner violence (IPV) during pregnancy and prevalence of gestational diabetes mellitus (GDM) included.

	Univariate Analysis			Multivariate Analysis		
	Odds Ratio	95% Confidence Interval	*p*-Value	Adjusted Odds Ratio	95% Confidence Interval	*p*-Value
Dependent variable: Corresponding author female (Logistic regression Reference group: male)						
Type of study (Reference group: IPV)	1.11	0.68; 1.83	0.669	1.16	0.70; 1.93	0.570
Human Development Index (Reference group: Low HDI) (*)	2.18	1.20; 4.00	0.011	2.24	1.22; 4.10	0.009
Journal impact factor (Reference group: Low JIF) (**)	0.65	0.39; 1.09	0.104	0.62	0.37; 1.05	0.075
Year (Reference group: <2015)	0.76	0.46; 1.25	0.280	-	-	-

(*) Low HDI versus the rest. (**) Q3, Q4 and not indexed versus Q1 and Q2.

## Data Availability

Not applicable.
